# Microaspiration of *Solanum tuberosum* root cells at early stages of infection by *Globodera pallida*

**DOI:** 10.1186/s13007-017-0219-x

**Published:** 2017-08-24

**Authors:** Rinu Kooliyottil, Louise-Marie Dandurand, Joseph C. Kuhl, Allan Caplan, Fangming Xiao

**Affiliations:** Department of Plant Soil and Entomological Sciences, University of Idaho, Moscow, ID 83844 USA

**Keywords:** Plant parasitic nematode, Feeding site, Microaspiration, Transcriptome, Pathogenesis related genes

## Abstract

**Background:**

Sedentary endoparasitic cyst nematodes form a feeding structure in plant roots, called a syncytium. Syncytium formation involves extensive transcriptional modifications, which leads to cell modifications such as increased cytoplasmic streaming, enlarged nuclei, increased numbers of organelles, and replacement of a central vacuole by many small vacuoles. When whole root RNA is isolated and analyzed, transcript changes manifested in the infected plant cells are overshadowed by gene expression from cells of the entire root system. Use of microaspiration allows isolation of the content of nematode infected cells from a heterogeneous cell population. However, one challenge with this method is identifying the nematode infected cells under the microscope at early stages of infection. This problem was addressed by staining nematode juveniles with a fluorescent dye prior to infection so that the infected cells could be located and microaspirated.

**Results:**

In the present study, we used the fluorescent vital stain PKH26 coupled with a micro-rhizosphere chamber to locate the infected nematode *Globodera pallida* in *Solanum tuberosum* root cells. This enabled microaspiration of nematode-infected root cells during the early stages of parasitism. To study the transcriptional events occurring in these cells, an RNA isolation method from microaspirated samples was optimized, and subsequently the RNA was purified using magnetic beads. With this method, we obtained an RNA quality number of 7.8. For transcriptome studies, cDNA was synthesized from the isolated RNA and assessed by successfully amplifying several pathogenesis related protein coding genes.

**Conclusion:**

The use of PKH26 stained nematode juveniles enabled early detection of nematode infected cells for microaspiration. To investigate transcriptional changes in low yielding RNA samples, bead-based RNA extraction procedures minimized RNA degradation and provided high quality RNA. This protocol provides a robust procedure to analyze gene expression in nematode-infected cells.

## Background

*Globodera pallida* (Stone) Behrens (pale cyst nematode, PCN) is a sedentary endoparasitic nematode that causes severe yield loss of potatoes (*Solanum tuberosum* L.). Infective second stage juveniles (J2s) of *G. pallida* invade the roots of the host plant and establish a feeding site by fusing several cells into a single syncytium [10, 15, 22, 28]. The nematode secretes a cocktail of proteins called effectors which are responsible for altering the cellular machinery of susceptible plants leading to the formation of the syncytium [6, 16]. Understanding how host gene expression changes after *G. pallida* infection is important for the development of resistance as is needed for potato including russet-type varieties.

Genes upregulated in nematode infected plants have been classified into primary (genes induced up to 1-day post infection) or secondary upregulated genes (genes induced after 3-days infection) [25]. Primary upregulated genes may be involved in wounding, pathogen recognition, and syncytium initiation, whereas, many of the genes induced later have been associated with syncytial development and maintenance. Genes responding to stress or pathogen defenses, involved in the cell cycle, or associated with the cytoskeleton fall into both categories [25]. Swiecicka et al. [25] reported 20% greater expression of genes associated with phytohormone responses in the primary category compared to the secondary category in *Globodera rostochiensis*-infected *Solanum lycopersicum*. On the other hand, Ithal et al. [7] found a significantly higher expression of genes related to defense responses 2–5 days after *Glycine max* roots were infected by *Heterodera glycines*. In a recent study, we have reported localized cell death as early as 2 days post infection in *Solanum sisymbriifolium* roots [14]. In the present study, we developed a methodology to study cell-specific transcription in potato roots during early *G. pallida* infection stages.

In recent years, several investigators have conducted gene expression studies from nematode infected roots [2, 12, 15]. In most of these experiments, total RNA was isolated from the entire root system of an infected plant. The nematode infected plant cells are only a small fraction of the total root material, and as a result, changes in expression in a few cells at the site of infection are likely to be overshadowed by potentially less relevant changes induced throughout the root. In order to identify changes in gene expression occurring only in the nematode-infected plant cells, many researchers collect cellular materials from infected cells using either laser capture microdissection (LCM) [1, 9, 19] or microaspiration [26, 29]. Ramsay et al. [19] used LCM to study the expression of cell cycle genes in the cytoplasm of giant cells isolated from tomato roots infected with the root knot nematode *Meloidogyne javanica* 4 days post infection. Anjam et al. [1] successfully standardized a method for LCM sample processing for transcriptome analysis of *Arabidopsis* roots infected by *Heterodera schachtii* and compared pathogenesis related gene expression in 5-day-old syncytial cells to uninfected cells using qPCR.

At early stages of infection, nematode infected plant cells are structurally similar to other cells in the root system which makes them difficult to differentiate using microscopy techniques. To alleviate difficulties in recognition of infected tissue, many investigations on cell specific transcriptome analyses of nematode infected plant cells have been performed on tissues in later stages of parasitism when cells are morphologically distinguishable. For instance, to investigate the differential expression of several pathogenesis related genes, Wang et al. [29] microaspirated giant cells formed by *M. javanica* in tomato roots 25 days post infestation. For studies of 5 day old *H. schachtii* infected *Arabidopsis* cells, Szakasits et al. [26] found a clear difference in gene expression between syncytia and other root cells using microaspiration. In the present study, our effort was to develop a methodology to locate the nematode inside the plant roots at early stages of parasitism during the initiation of a syncytium, and to isolate the content of these cells for RNA isolation. PKH26, a red fluorescent cell linker that binds to cell membrane lipids has been used for observation of plant parasitic nematodes when infecting plant roots [4, 14, 24]. In the present study, we have used this dye to select nematode-infected cells in *S. tuberosum* roots that were grown in micro-rhizosphere chambers (micro-ROCs). In addition, a protocol was optimized for RNA isolation from microaspirated samples and utilized for downstream applications such as qPCR analyses.

## Results

### PKH26 fluorescent labelling coupled with micro-ROCs for microaspiration of *Globodera pallida* infected plant cells

To investigate the cell specific transcriptome of nematode infected plant cells at early stages of infection, we developed a non-destructive microaspiration method. Plants were grown in micro-ROCs, which allows non-destructive detection and observation of nematodes within plant roots [14]. To prevent movement, desiccation and wounding of the root samples while microapsirating, plants were transferred from the micro-ROC to a glass slide lined with agarose.

**Fig. 1:**
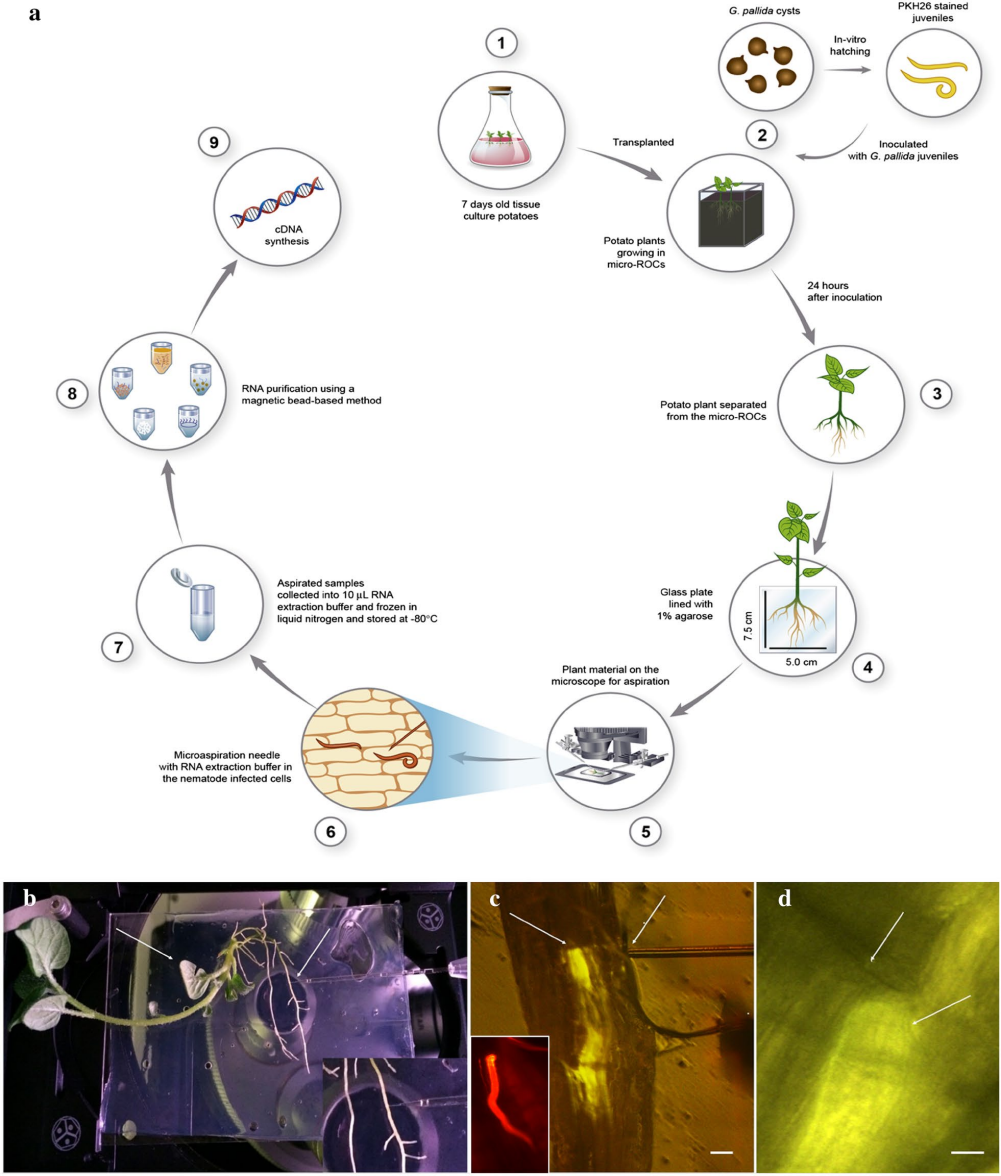
Process of microaspiration and RNA isolation from *Globodera pallida* infected *Solanum tuberosum* root cells. **a** Schematic diagram of the workflow of nematode inoculation, microaspiration of infected cells and RNA isolation. **b** *S*. *tuberosum* on a glass plate (7.5 cm L × 5.0 cm W) ready for microaspiration, the *left arrow* shows the glass plate lined with agarose; *right arrow* shows the needle and the root; the *inset* is of a close view of the root perforated by the capillary needle. **c** PKH26 stained *G*. *pallida* located under a fluorescent microscope with capillary needle inside the root cell; the bright field illumination was turned on to a low setting to allow the capillary needle to be seen; the *inset* shows the image of *G. pallida* through a rhodamine filter without bright field illumination; the *left arrow* indicates the head region of the nematode; *right arrow* shows the root perforated by the capillary needle. **d** The head region of *G. pallida* (*lower arrow*) inside the root cell with the aspirator needle (*upper arrow*). The needle contains extraction buffer to prevent RNA degradation. *Bar* = 20 µM

In the past, locating infected plant cells to study cellular content has been difficult because detection methods were not available. To facilitate this process, hatched J2s were stained with PKH26 prior to inoculation on their host. With a rhodamine filter (dye excitation max. 551 nm and emission max. 567), PKH26 stained nematodes fluoresce red, and their movement and position was easily observed. To microaspirate cell content both nematode and plant cells need to be visualized at the the same time. To achieve this, brightfield was turned to a low setting which allowed visualization of the plant cell while also maintaining the fluorescence to visualize the site of the nematode within the plant root (Fig. 1). In Fig. 1c and the nematode is yellow due to the bright field illumination of the microscope which was turned on to enable visualization of the capillary needle for microaspiration. An image of PKH26 stained *G. pallida* with the rhodamine filter (in the absence of bright field illumination) has been inserted in Fig. 1c. Under these conditions, the capillary needle was visible and could be directed to the cells infected by the nematode. Until we filled the capillary needle with RNA extraction buffer (ARCTURUS^®^ PicoPure^®^ RNA isolation kit) to prevent RNA degradation, our early efforts resulted in degradation of the microaspirated RNA. Fragment analysis revealed higher levels of RNA once the capillary needle was filled with buffer prior to aspiration of cell content.

### Standardization of RNA isolation protocol from microaspirated plant cells

**Fig. 2:**
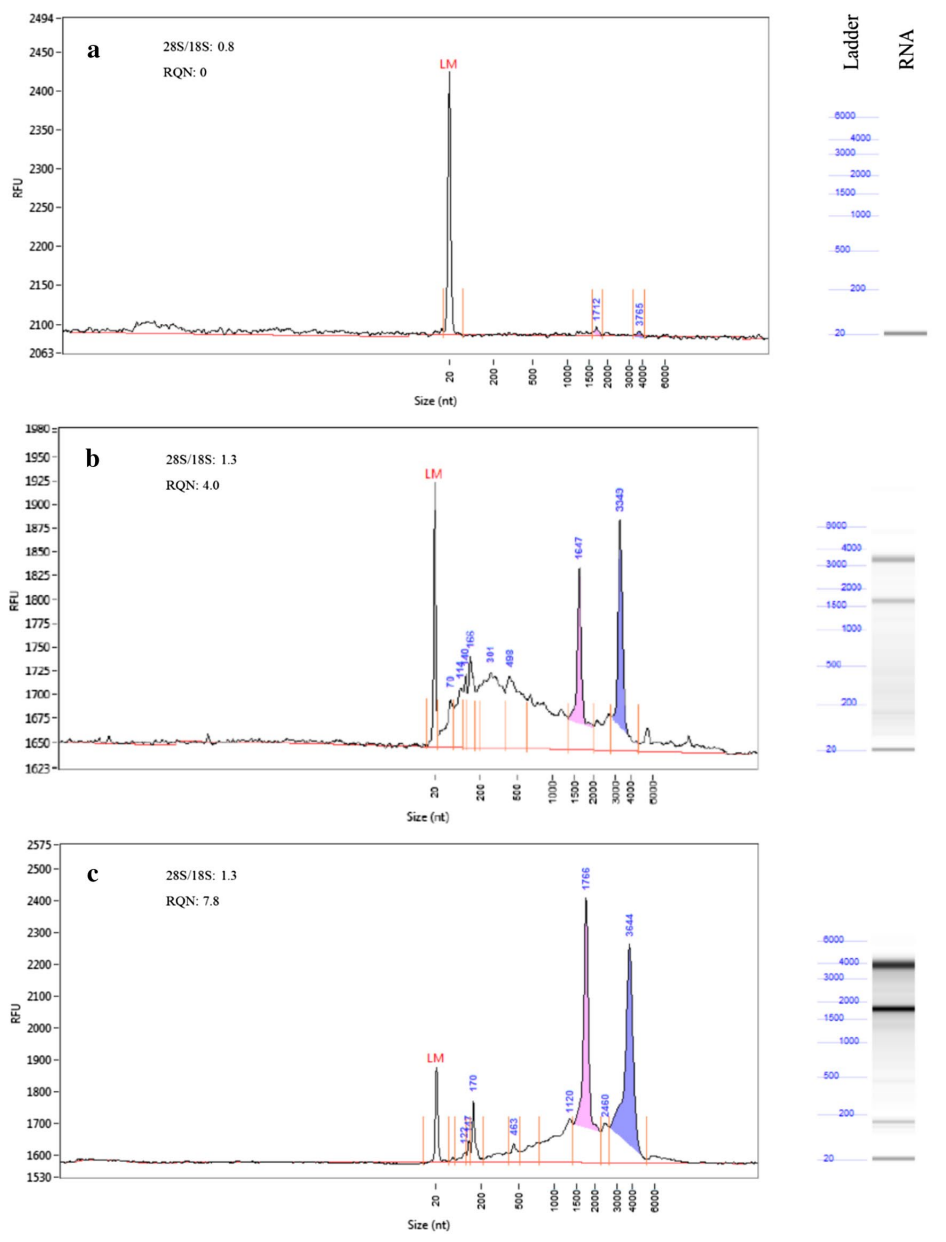
Quality assessment of RNA isolated from microaspirated samples from *Globodera pallida* infected potato cells. **a** RNA isolated using Picopure RNA isolation kit protocol from samples incubated at 42 °C for 30 min, the RNA quality number (RQN) number was 0; **b** Picopure RNA isolation kit protocol from samples incubated at 42 °C for 15 min, the RQN number was 4.0; **c** RNA isolated using magnetic bead-based method of Agencourt RNAdvance Tissue total RNA extraction Kit, the RQN number was 7.8. The quality assessment was performed on a fragment analyzer

Wounding of plant cells causes expression of wound-inducible genes. To minimize wounding effect from the root samples, only one sample was aspirated from each root. Initially, the quality and quantity of RNA extracted and purified from this small amount of sample using the ARCTURUS^®^ PicoPure^®^ RNA isolation kit protocol was not adequate for further downstream processes (Fig. 2). To increase the purity and quality of RNA from the microaspirated samples, we modified the ARCTURUS^®^ PicoPure^®^ RNA isolation kit protocol by reducing the initial incubation time from the suggested 30 min by 5 min increments from 0 to 20 min. Incubating the sample for 15 min provided the highest quality RNA, with the RNA Quality Number (RQN) of 4.0 and the total concentration of 0.85 ng/µL (Fig. 2). To further improve the quality of RNA, the sample was further processed by using a magnetic bead-based protocol. This method yielded 12.8 ng RNA with an RQN of 7.8 dissolved in 10 µl (Fig. 2), or a total of 12.8 ng per aspirated sample.

### Amplification of genes from *Globodera pallida* infected *Solanum tuberosum* cells

To test the downstream applications of the RNA isolated from nematode-infected plant cells, cDNA was synthesized, and one housekeeping gene, elongation factor, and 4 pathogenesis related (PR) genes (genes contributing to disease resistance), NADH-quinone oxidoreductase subunit C, CHTB4 (Chitinase), cinnamyl alcohol dehydrogenase, and 6-phosphogluconate dehydrogenase, were amplified using specific primers (Table 1). The PR genes were selected randomly from our RNAseq data (data not shown).

**Table 1:** Primer sequences used for amplification of cDNA samples synthesized from RNA isolated from microaspirated *Globodera pallida* infected and un-infected potato roots cells

Gene	Primer sequence (5′–3′)	Product size (bp)	Genbank accession number
Elongation factor	Forward AACCGCTGAGAACTTCCGAG Reverse CGACCCAACAAGACACAAGC	566	AJ536671.1
Cinnamyl alcohol dehydrogenase	Forward TGCACATAACAGGGGAGCTG Reverse ATTACAGAGGCGCAACCAGG	579	JQ619511.1
6-Phosphogluconate dehydrogenase	Forward TTGGTGGGAGGTTTCTCGTG Reverse CCTGCCAATTTGGTTCAGGC	502	XM_006363489.2
NADH-quinone oxidoreductase subunit C	Forward CCAAGACTAAGGCCGAGCAA Reverse GGACGGATCACCTGTCGATC	520	AY056140.1
CHTB4 (Chitinase)	Forward CTGTTGCAGCAATTTCGGCT Reverse AAATTTGGATGGGGCCTCGT	576	U02608.1

Except for elongation factor, all primers were designed using gene sequences from RNAseq data. Therefore, the Genbank accession numbers given here are of identical sequences found in the NCBI

**Fig. 3:**
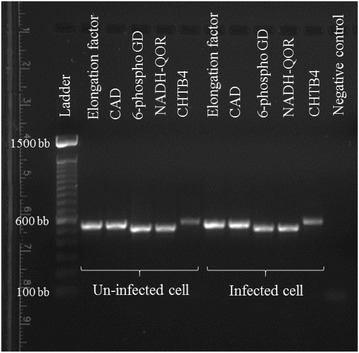
PCR Analysis of housekeeping (elongation factor) and pathogenesis related (*CAD* Cinnamyl alcohol dehydrogenase, *6-phospho GD* 6-phosphogluconate dehydrogenase, *NADH-QOR* NADH-quinone oxidoreductase subunit C, *CHTB4* Chitinase) genes in *Globodera pallida* infected and uninfected root cells of potato. Ladder is 100–1500 bp

All genes were successfully amplified from the microaspirated cDNA derived samples and clear bands were observed on the electrophoresis gel (Fig. 3). Nematode infected plant cells were compared with non-infected cells for gene expression. qPCR analysis of the CHTB4 gene from cDNA 3 independent pairs of infected and uninfected samples showed 1.15 times higher expression of CHTB4 in infected cells compared to uninfected cells 24 h post infestation [cycle threshold (Ct) of CHTB4 in infected cell cDNA = 23.76 ± 0.04, Ct of CHTB4 in control cell cDNA = 23.66 ± 0.02, Ct of elongation factor in infected cell cDNA = 21.35 ± 0.03, and Ct of elongation factor in control cell cDNA = 21.04 ± 0.04]. This indicates that RNA isolated from the micro aspirated samples can be utilized for transcriptome analysis of nematode infected plants. RNAseq libraries were also prepared from the RNA isolated in this study but the comparative transcriptomics results will be presented elsewhere.

## Discussion

Cell specific transcriptome analysis using microaspiration and LCM have been successfully used to isolate cellular materials from the syncytium for plant-nematode interaction studies [1, 9, 18]. Tissue samples for transcriptome analysis obtained by LCM need to be fixed prior to sectioning and mounting [1, 3], which can cause RNA degradation.

Despite being an inexpensive technique compared to LCM, microaspiration is laborious for obtaining sufficient amounts of input sample for RNA isolation [1], and can only be performed once the syncytium is morphologically distinct. To overcome these limitations, we standardized a microaspiration method for early detection of infection sites, by staining the nematodes using a fluorescent stain, modifying incubation time and by using a bead-based RNA purification protocol. With these modifications, we obtained high quality RNA from low input samples for transcriptome analyses.

Recent studies using microscopy assisted techniques are conducted, in most cases 5 days or later after infection [1, 9, 11, 19, 26, 29]. The main constraint in isolating cells of interest at early stages is in visualizing the nematode and the nematode infected plant cells prior to morphological changes caused by formation of the syncytium which is typically 4–5 days post infection. The present study reports an improved microaspiration method for early stages of infection.

To study the nematode-plant interaction, we used PKH26 coupled with micro-ROCs [4, 14], which allows observation of live nematode inside the plant roots. The plants grown in micro-ROCs were removed with minimal damage to roots. To prevent slipping of the samples and desiccation or wounding of the roots, we used a glass plate layered with agarose. Wounding induces defense proteins [5, 21]. To reduce these effects, we aspirated the contents of one cell from each root system. To obtain sufficient RNA, others, such as Juergensen et al. [11], aspirated 100 nematode infected cells from each root system for transcriptome analysis.

After aspirating the cell contents from infected cells, we attempted several methods to isolate pure RNA from this sample. Although, the ARCTURUS^®^ PicoPure^®^ RNA isolation kit has been used for isolation of RNA from low yielding samples [1, 8, 19], no mention of RNA quality obtained from these sample was made. Anjam et al. [1] standardized the LCM sample fixation protocol and reported the RNA integrity number of 7.8 using the ARCTURUS^®^ PicoPure^®^ RNA isolation kit. Despite several attempts using the manufacture’s protocol for the ARCTURUS^®^ PicoPure^®^ RNA isolation kit we did not get sufficient amounts or quality of RNA for downstream applications. However, when we reduced the incubation time from the initial 30 to 15 min an RQN of 4.0 was obtained. Most of the commercially available kits use column separation methods, which, in our experiments, did not yield high quality RNA for downstream applications such as RNAseq. In order to obtain RNA of adequate quality, we replaced columns with magnetic beads (Agencourt RNAdvance Tissue total RNA extraction kit) and were able to obtain RQN values of 7.8. Karrer et al. [13] used oligo (dT)-linked magnetic beads for cDNA synthesis directly from aspirated samples. In magnetic bead-based protocols, nucleic acids present in the solution are first bound to a magnetic carrier that have immobilized affinity ligands and then washed with a buffer to remove contaminants, and finally eluted using nuclease free water [27]. In our study, this method gave promising quantities and improved the quality of RNA from low amounts of input material.

To evaluate whether the RNA we obtained could be used for downstream applications, the isolated RNA was converted into cDNA and used for RNAseq and qPCR. Elongation factor and PR protein coding genes were amplified from these cDNA samples. qPCR was also successfully conducted for gene expression analysis from infected and non-infected cells of the same plant. RNAseq libraries were also prepared from this study and the results will be published elsewhere.

## Conclusion

Because of the ability to study a single cell-type from a heterogeneous population, cell specific transcriptome analysis has become increasingly popular in animal, human and plant systems. Sedentary endoparasitic nematodes, such as cyst and root knot nematodes, form a feeding structure in the vascular tissues of the plant roots, and these tissues are metabolically different from other plant tissues due to the interaction between the host and the pathogen. It is crucial to understand the nematode-plant interaction at early stages of infection because early responses that may be suppressed in later stages, may be important for parasitism and host defense. PKH26 stained juveniles were used to locate infected sites for microaspiration. The RNA quality was improved significantly by adjusting the incubation time of the ARCTURUS^®^ PicoPure^®^ RNA isolation kit and by using a bead-based RNA purification kit (Agencourt RNAdvance Tissue total RNA extraction kit). As evidenced by qPCR of genes from infected and non-infected material, this RNA was of sufficient quality for gene expression studies. The approach presented here will provide suitable methods for early parasitism studies in nematode-plant interactions at the cellular level.

## Methods

### Plant material and nematode inoculum

Planting, nematode inoculum preparation and inoculation were performed as described in Kooliyottil et al. [14]. In brief, 1-week old *Solanum tuberosum* cv. ‘Desiree’ grown in the tissue culture medium was transplanted into micro-ROCs and kept under greenhouse conditions (18 ± 2 °C, 16 h light and 8 h dark cycle). *Globodera pallida* encysted eggs were hatched in potato root diffusate. The hatched second stage juveniles (J2s) were surface sterilized and stained using PKH26 (Sigma Aldrich, USA) as per the manufaturer’s protocol, and then suspended in 0.01% sterile agarose. Two weeks after planting in micro-ROCs, pproximately 200 J2s suspended in agarose were inoculated onto the roots of *S. tuberosum* [4, 14].

### Microaspiration of *G. pallida* infected cells

Twenty-four hours after inoculation, the plant was carefully removed from the micro-ROCs by breaking the glass plate and gently washing the roots with sterile water for 15 s. The plant was then placed on to a glass plate (7.5 cm L × 5.0 cm W) previously lined with 1% agarose (Fig. 1a). Infected roots were observed with a fluorescent microscope (DMI 3000B, Leica, Germany) and fluorescing *G. pallida* J2s were located within the root by use of a rhodamine filter under low magnification (20X objective lens).

To aspirate cell content, the capillary needle (TransferTip^®^—R ICIS, inner diameter 4 µM, outer diameter 7 µM, tip angle 35°, Eppendorf, Hamburg, Germany) of the micro manipulator (CellTram^®^ vario, Eppendorf, Hamburg, Germany) filled with RNA extraction buffer (ARCTURUS PicoPure RNA Isolation Kit, Life Technologies, USA) was used to pierce the plant root with assistance from TransferMan^®^ (Eppendorf, Hamburg, Germany). While entering the root, the CellTram^®^ vario was turned to the injection mode to avoid the entry of any unwanted plant cell material into the needle due to the turgor pressure of the plant cell. Once the infected cell was reached, the CellTram^®^ vario was turned to the aspiration mode to aspirate plant cellular material. The microaspirate contents were immediately transferred to 10 µL RNA extraction buffer provided in the ARCTURUS PicoPure RNA Isolation Kit (Life Technologies, USA), frozen in liquid nitrogen, and stored at −80 °C. Cell content of uninfected cells from the infected roots were also aspirated for gene expression comparison.

### RNA isolation from microaspirated plant material, cDNA synthesis and gene amplification

Initial attempts to isolate total RNA from the microaspirated cell content were conducted by using the manufacture’s protocol from ARCTURUS PicoPure RNA Isolation Kit (Life Technologies, USA). Frozen microaspirated samples were thawed by incubating at 42 °C for 30 min. However, the quality of RNA extracted in this way was not sufficient for further analysis despite several attempts. Reduction in the initial incubation time was attempted by first using 30 min (as recommended manufacture’s protocol), and then at intervals of 5 min from 0 to 20 min; 15 min interval provided the best quality RNA. However, to further improve the quality, samples were processed by using a magnetic bead-based method. In brief, the samples incubated at 42 °C for 15 min (from the above mentioned protocol), were supplemented with the magnetic bead buffer provided in the Agencourt RNAdvance Tissue total RNA extraction kit (Beckman Coulter, USA) and further steps of magnetic separation and washing was performed as per the protocol provided by the manufacturer. To avoid any genomic DNA contamination in the RNA, each sample was treated with DNAse following the recommended protocol from the Agencourt RNAdvance Tissue total RNA extraction kit.

Qualitative and quantitative analysis of the purified RNA was determined by using a FRAGMENT ANALYZER™ (Advanced Analytical Technologies Inc., USA). RNA was reverse-transcribed into cDNA according to the procedure described in the SUPERSCRIPT^®^ II reverse transcriptase (Invitrogen) using oligo (dT) primer (Thermo Fisher Scientific Inc, MA, USA) and dNTP mix. The quality and quantity of cDNA was determined by using a NanoDrop™ 2000c instrument (Thermo Scientific Inc, MA, USA). The cDNA from infected and uninfected samples were amplified using conventional PCR, with a housekeeping gene specific primer for elongation factor, and pathogenesis related (PR) genes namely NADH-quinone oxidoreductase subunit C, CHTB4 (Chitinase), and 6-phosphogluconate dehydrogenase [17, 20, 23]. The PCR (C1000™ Thermal Cycler, BIO-RAD, USA) conditions were as follows: Initial denaturation, 95 °C for 3 min; denaturation, 95 °C for 30 s; 62 °C for 30 s; 72 °C for 1:30 min; total cycles 40; final extension, 72 °C for 5 min. The specific primers used for the amplification are given in Table 1.

To confirm gene expression levels in microaspirated infected and uninfected cells, cDNA from both samples were analyzed for expression of CHTB4 using qPCR (ViiA™ 7 Real-Time PCR System, Applied Biosystems, USA). The reaction was prepared in a total of 20 µL in which 10 µL master mix (FAST SYBR™ Green Master Mix, Thermo Fisher Scientific Inc, MA, USA), 1 µL template cDNA (10 ng), 150 nM (primer efficiency = 102.30, R^2^ = 0.99) each of forward and reverse primers of elongation factor and 100 nM (primer efficiency = 103.85, R^2^ = 0.97) each of forward and reverse primers of CHTB4, and nuclease free water was added. The PCR condition was as follows: 50 °C for 2 min, 95 °C for 10 min, 60 °C for 1 min, 95 °C for 15 s 15 °C for 1 min and 95 °C for 15 s with a total of 40 cycles. The relative expression level of CHTB4 was calculated in comparison to elongation factor by means of 2^(control sample Ct of target−infected sample Ct of target)^ ÷ 2^(control sample Ct of reference−infected sample Ct of reference)^. The results presented are the average of three independent infections and sample collections.
